# The importance of manual white blood cell differential counts and platelet estimates in elephant hematology: blood film review is essential

**DOI:** 10.1080/01652176.2020.1867329

**Published:** 2021-01-14

**Authors:** Tatiana C. Weisbrod, Ramiro Isaza, Carolyn Cray, Laurie Adler, Nicole I. Stacy

**Affiliations:** a Department of Comparative, Diagnostic, and Population Medicine, College of Veterinary Medicine, University of Florida, Gainesville, FL, USA; b Department of Large Animal Clinical Sciences, College of Veterinary Medicine, University of Florida, Gainesville, FL, USA; c Department of Pathology & Laboratory Medicine, Miller School of Medicine, University of Miami, Miami, FL, USA

**Keywords:** African elephant, Asian elephant, complete blood count, *Elephas maximus*, *Loxodonta africana*, hematology

## Abstract

Unique features of elephant hematology are known challenges in analytical methodology like two types of monocytes typical for members of the Order Afrotheria and platelet counts of the comparatively small elephant platelet. To investigate WBC differential and platelet data generated by an impedance-based hematology analyzer without availability of validated species-specific software for recognition of elephant WBCs and platelets, compared to manual blood film review. Blood samples preserved in ethylenediaminetetraacetic acid (EDTA) of 50 elephants (n = 35 *Elephas maximus* and n = 15 *Loxodonta africana*) were used. A Mann-Whitney test for independent samples was used to compare parameters between methods and agreement was tested using Bland-Altman bias plots. All hematological variables, including absolute numbers of heterophils, lymphocytes, monocytes, eosinophils, basophils, and platelets, were significantly different (*p* < 0.0001) between both methods of analysis, and there was no agreement using Bland-Altman bias plots. Manual review consistently produced higher heterophil and monocyte counts as well as platelet estimates, while the automated analyzer produced higher lymphocyte, eosinophil, and basophil counts. The hematology analyzer did not properly differentiate elephant lymphocytes and monocytes, and did not accurately count elephant platelets. These findings emphasize the importance of manual blood film review as part of elephant complete blood counts in both clinical and research settings and as a basis for the development of hematological reference intervals.

## 1. Introduction

The complete blood count (CBC) is a fundamental component of the minimum diagnostic database for elephants under managed care. African (*Loxodonta africana*) and Asian *(Elephas maximus*) elephants possess unique leukocyte morphology which can make differentiation of cell types challenging. Similar to other members of the *Afrotheria* including manatees and hyraxes, elephants have heterophils rather than neutrophils and two distinct monocyte morphologies (Aroch et al. 2007; Harr et al. 2010). In addition to the typical monocyte type with a more rounded to reniform nucleus commonly seen in blood films of domestic mammalian species, elephants also have a monocyte type with a bi-lobed nucleus that can rarely be tri-lobed (Allen et al. 1985; Harr et al. 2010). These bi-lobed monocytes have peroxidase-positive staining characteristics of their cytoplasm which indicates phagocytic function; this finding supports monocytic rather than lymphocytic cell lineage of the unique bi-lobed cell (Harr et al. 2010). The morphological similarity of bi-lobed monocytes to lymphocytes has historically resulted in misidentification leading to over-reporting of lymphocyte and under-reporting of monocyte proportions in the elephant hematology literature (Harr et al. 2010). The rare tri-lobed monocytes can readily be differentiated from lymphocytes due to their unique nuclear morphology.

Proper identification of white blood cell (WBC) types is the basis for an accurate leukocyte differential count. Automated hematology analyzers are commonly utilized for CBC processing in domestic mammalian species and can be less labor-intensive and more precise than manual methods (Kjelgaard-Hansen and Jensen 2006). It has been speculated that the morphological similarities between elephant lymphocytes and bi-lobed monocytes may result in imprecise differentiation by automated analyzers; however, no information regarding automated analyzer accuracy is available to date (Stacy et al. 2017). Automated analyzers rely principally on electrical impedance analysis or laser flow cytometry analysis for differentiation of various blood constituents. Impedance-based analyzers differentiate WBC types based on electrical interferences of cells flowing through a conductive liquid as they pass through an aperture, while flow cytometric analyzers use laser light scatter to determine differences in cellular size, internal structure, granularity, and surface structure (DeNicola 2011).

The objective of this study was to investigate WBC differential and platelet data generated by an impedance-based hematology analyzer without availability of validated species-specific software for recognition of elephant WBCs and platelets, compared to manual blood film review. Our null hypothesis was that there would be no significant differences between WBC differentials or platelet estimates as determined by both analytical methodologies.

## 2. Materials and methods

Blood samples were collected from 35 Asian elephants (*Elephas maximus*) and 15 African elephants (*Loxodonta africana*) for a total of 50 individual elephants located at multiple zoological institutions throughout the USA. All blood samples were obtained during collection for routine wellness or clinical disease monitoring following standard venipuncture techniques at the direction of attending veterinary staff. Blood was preserved in ethylenediaminetetraacetic acid (EDTA) vacutainer collection tubes and two fresh blood smears were created within two hours of sample collection. EDTA-preserved whole blood samples and air-dried blood films were packaged and shipped on cold packs overnight to the Avian & Wildlife Laboratory at the University of Miami, Florida, USA. The samples were analyzed upon receipt.

Blood samples were processed using the Hemavet 1700FS (Drew Scientific Inc., Miami Lakes, FL, USA), an impedance-based automated hematology analyzer without availability of validated species-specific commercial software for recognition of elephant leukocytes and platelets. This analyzer was selected based on its availability and routine use for veterinary blood samples at the diagnostic laboratory. The analyzer was maintained according to manufacturer specifications inclusive of daily quality control analysis. For this project, the elephant setting was utilized. It is acknowledged that while the setting is available on this instrument, it has not been validated for its use. The automated analyzer provided a WBC differential and was utilized for determination of the total WBC count which was used for calculations of absolute WBC concentrations for both WBC differential techniques, automated and manual. For manual blood film review, blood films were stained with Wright-Giemsa (Harleco®, EMD Millipore, Billerica, MA, USA) prior to evaluation. Blood films were only evaluated and included in this study if they were considered of adequate quality for blood film review by general laboratory standards. White blood cell morphology was carefully evaluated under ×100 objective for a 200 WBC manual differential. Platelet estimates (platelets per microliter) were performed by counting the platelets in one ×100 objective field multiplied by 15,000, which is a combination of two methods, since elephants often have >1,000 K/µl platelets with inflammatory conditions (i.e. inflammatory thrombocytosis; per observation by the authors) (Cornell University College of Veterinary Medicine; Harvey 2012). In blood films with <20 platelets per objective field, it is recommended to review 10 objective fields, calculate the average, and multiply by 15,000 (Cornell University College of Veterinary Medicine). All blood film reviews were performed by one investigator (NS).

A Mann-Whitney test for independent samples was used to compare parameters between methods and agreement was tested using Bland-Altman bias plots. All statistical analyses were performed using MedCalc statistical software (MedCalc, Version 19.2.1, Copyright 1993-2020, MedCalc Software Ltd, Ostend, Belgium).

## 3. Results

**Figure 1.**
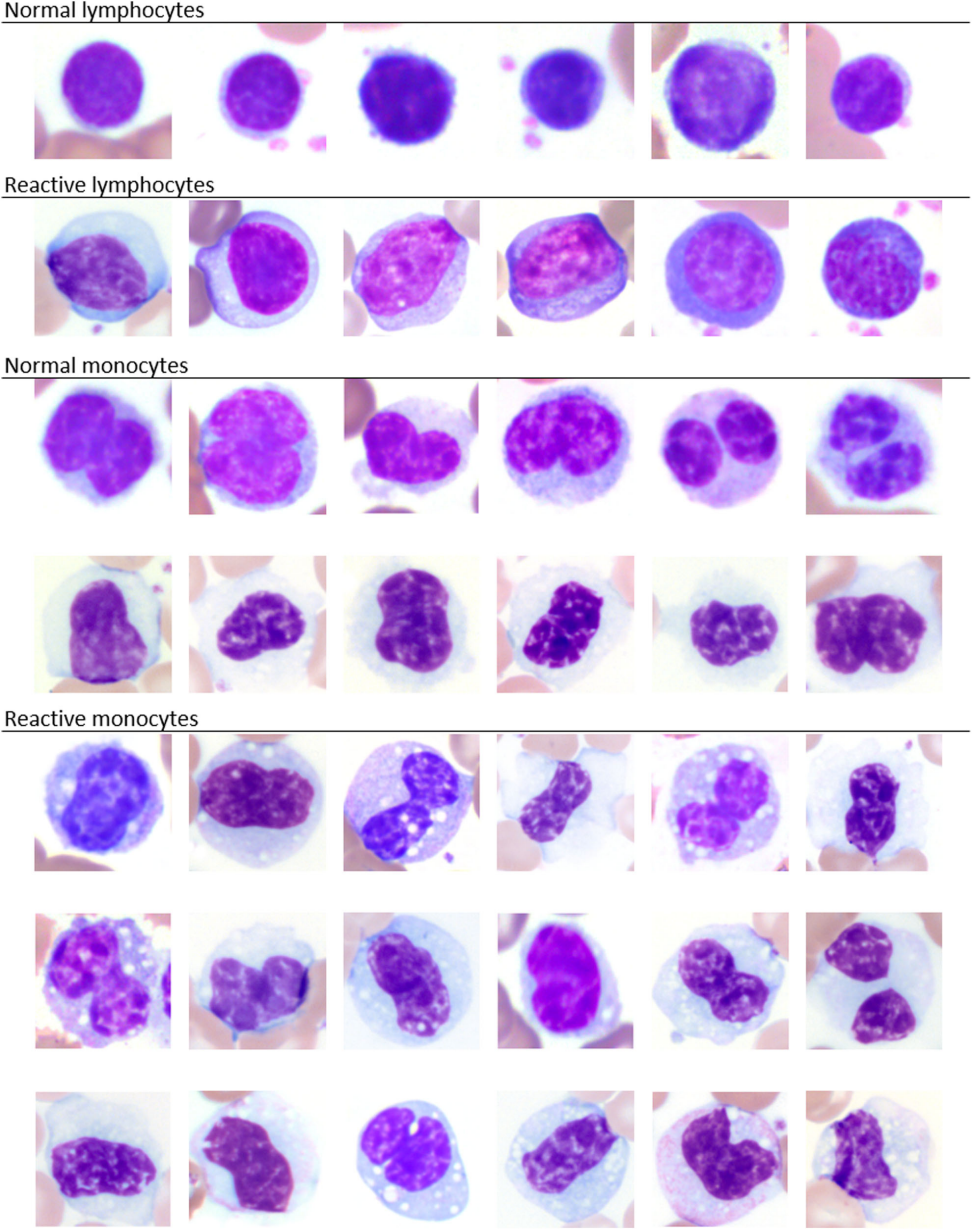
Image composite of normal and reactive lymphocytes and monocytes, respectively, in elephants as commonly recognized by blood film evaluation. x100 objective. Wright-Giemsa stain.

Thirty-six female and 14 male elephants were included in this study. Animal age ranged from 3 to 72 years. The clinical history was unknown with a presumed variety of samples from wellness examinations in addition to clinical cases as routinely submitted to the laboratory. This provided a range of concentrations of WBC and platelets for method comparisons. Examples for morphologies of normal versus reactive lymphocytes and monocytes, respectively, are given in Figure 1. The morphologies of activated lymphocytes and monocytes can occur with underlying inflammation and are generally considered non-specific in mammals (Harvey 2012). However, the morphologies could present an additional reason for the difficulty of differentiating lymphocytes and monocytes by hematology analyzer as well as by blood film review. Overt platelet clumping was not identified by blood film review.

**Figure 2.**
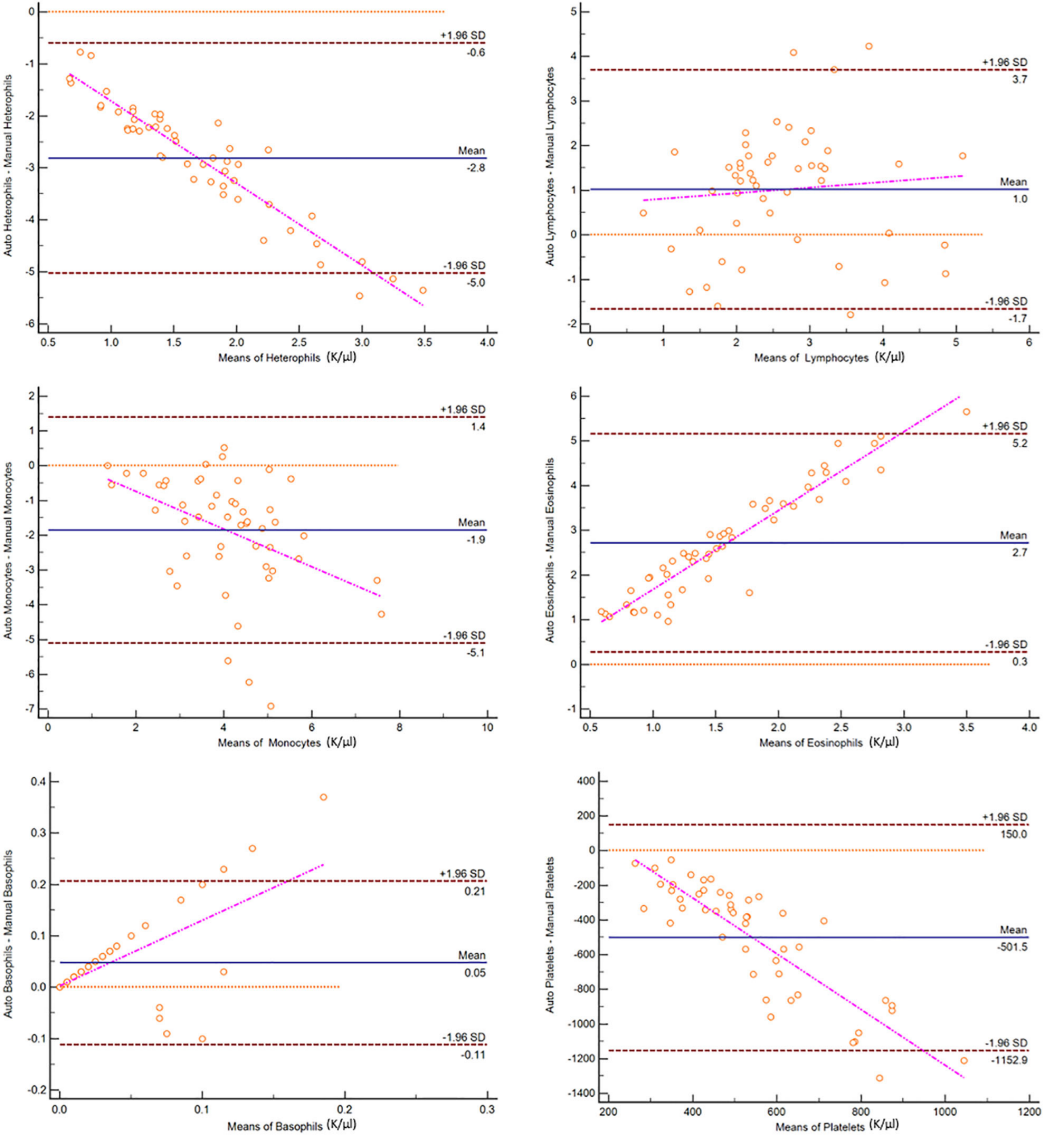
Bland-Altman plots for comparison of white blood cell and platelet quantification methods as determined using an automated hematology analyzer and manual blood film evaluation in 50 elephants. The difference between automated and manual cell counts is plotted against the mean count of both methods for each cell type. Central lines depict the mean difference between the two methods, upper and lower lines represent limits of agreement, defined as the mean difference ±1.96 times the standard deviation of the differences.

All hematological variables, including absolute numbers of heterophils, lymphocytes, monocytes, eosinophils, basophils, and platelets were significantly different (*p* < 0.0001) between the two methods of analysis (Table 1). The WBC differential by the automated analyzer produced significantly higher lymphocyte, eosinophil, and basophil counts while the manual blood film review produced significantly higher heterophil and monocyte counts, as well as platelet estimates. There was no agreement between methods based on Bland-Altman plots (Figure 2).

**Table 1.** Comparison of means for hematological analytes of 50 elephants between automated (A) versus manual (M) white blood cell differential, and automated platelet count versus platelet estimate from the blood film.

Hematological analyte	Method	Mean	Standard deviation	Range	p value
Heterophils (K/µl)	A	0.29	0.23	0.00 − 0.93	
	M	3.1	1.22	1.14 − 6.16	<0.0001
Lymphocytes (K/µl)	A	3.13	1.24	0.72 − 5.97	
	M	2.11	1.14	0.22 − 5.29	<0.0001
Monocytes (K/µl)	A	3.14	1.20	1.17 − 5.84	
	M	4.99	1.80	1.35 − 9.72	<0.0001
Eosinophils (K/µl)	A	2.94	1.27	1.18 − 6.33	
	M	0.23	0.21	0.00 − 0.97	<0.0001
Basophils (K/µl)	A	0.06	0.07	0.00 − 0.37	
	M	0.01	0.03	0.00 − 0.15	<0.0001
Platelets (K/µl)	A	294	91	106 − 509	
	M	793	333	300 − 1650	<0.0001

## 4. Discussion

This study documents the discrepancy of WBC differential and platelet counts by an impedance-based hematology analyzer compared to manual blood film review in Asian and African elephants. The automated hematology analyzer did not provide an accurate WBC differential mainly due to incorrectly differentiating between lymphocytes and monocytes. The key finding of this study is the consistently reported higher lymphocyte count than compared to manual blood film review, while the monocyte count was consistently higher by blood film review. This cellular differentiation is a widely known challenge of elephant hematology and reinforces the need for blood film review as an essential part of the CBC and for those reviewing blood films from elephants, to become familiar with the unique monocyte morphology in this species. The automated analyzer also appears to be challenged by accurately differentiating granulocytes, since basophils and eosinophils were higher than by blood film review. Accurate WBC differentials are essential for the development of reliable reference intervals, development of individual animal baselines, valid comparative clinical pathology applications, and are important in both clinical and research settings. Routine CBC monitoring has revealed that leukopenia characterized by monocytopenia and thrombocytopenia can be predictive of early infection with elephant endotheliotropic herpesvirus (EEHV) (Richman et al. 2000; Richman and Hayward 2012; Fuery et al. 2016; 2016). Early detection of these leukogram changes may facilitate quicker medical interventions as PCR results for detection of the virus may take up to 24 hours. Further, monitoring hemogram trends during EEHV infections are reportedly useful for guiding therapeutic decisions and gauging convalescence (Wissink-Argilaga et al. 2019). Adequate WBC recognition and their morphological changes in disease have been described with various causes of inflammation in elephants (Stacy et al. 2017). Inaccurate WBC differentials will result in incorrect calculations of absolute proportions of WBC types and thus have the potential to affect clinical decisions.

The automated analyzer did not accurately quantitate elephant platelets, presumably due to their small size, and resulted in consistently lower platelet counts compared to the estimates by blood film review. Accurate platelet estimates are important clinically and may be challenging to obtain in elephants as they are small and readily activated which can result in clumping (Plessis and Stevens 2002; Stacy and Hollinger 2018). Inflammation and neoplasia are the most common causes of thrombocytosis in dogs (Neel et al. 2012). Detection of thrombocytosis may be particularly relevant in species such as elephants who may fail to show quantifiable WBC changes until late in the course of inflammation (Stacy et al. 2017) or as manatees who lack a robust WBC response in the face of inflammatory disease (Harr et al. 2006). In addition to elevated band heterophils and eosinophils, thrombocytosis as determined by platelet estimates has also been associated with *Mycobacterium tuberculosis* positive elephants (Harr et al. 2001). Also, the authors have observed high platelet estimates >1,000 K/µl in elephants with inflammatory disease. Furthermore, the observation of platelet concentrations in elephants over time is essential in detecting trends in health and in cases of EEHV, as thrombocytopenia is a hallmark of the infection (Richman et al. 2000). Future studies using platelet estimates are needed to understand platelet concentrations in health for the establishment of reference intervals and changes in disease.

Total white blood cell counts in mammalian species should always be performed by an automated hematology analyzer (Harvey 2012). This study highlights the need for blood film review as a vital component of the CBC in elephants since manual blood film review provides the superior WBC differential as a basis for the calculation of absolute WBC proportions and the superior platelet quantification method as compared to the automated analyzer. Blood film review can be performed as an in-house analysis by trained personnel for point-of-care information on individual elephants. Though slower and technically more demanding, blood film review provides a more reliable WBC differential than automated impedance-based analyzers in the absence of validated species-specific software programs. Additionally, blood film examination can provide morphological details of WBC in response to disease (e.g. heterophil left shift/toxic change, reactive lymphocytes, or activated monocytes), which may be observed despite a normal leukogram (Harvey 2012; Stacy et al. 2017) in an elephant patient and may directly impact clinical decision-making. Future studies validating species-specific software and evaluating the accuracy and reliability of other automated analytical hematology technologies such as optical or laser flow cytometers are warranted.
